# Understanding the relationship between illness perceptions of breast cancer and perceived risk in a sample of U.A.E. female university students: the role of comparative risk

**DOI:** 10.1186/s12905-022-01771-5

**Published:** 2022-05-25

**Authors:** Maria J. Figueiras, David Dias Neto, João Marôco

**Affiliations:** 1 Department of Psychology, College of Natural and Health Sciences, Zayed University, Abu Dhabi, United Arab Emirates; 2 APPsyCI - Applied Psychology Research Center Capabilities and Inclusion, ISPA - Instituto Universitário, Lisbon, Portugal; 3 William James Center for Research, ISPA - Instituto Universitário, Lisbon, Portugal

**Keywords:** Breast cancer, Illness perceptions, Risk perception, Comparative risk, Lay beliefs, Arab women

## Abstract

**Background:**

In the Middle East region, the incidence of breast cancer (BC) has substantially increased in the last years. Despite a considerable body of research about BC in Arab countries, how illness perceptions of healthy women about BC may influence risk perception is unknown.

**Methods:**

A cross-sectional survey was conducted on a sample of 298 young Emirati women. The measures included demographic information, illness perceptions, and risk perception. Descriptive and correlational analyses were performed to assess illness perceptions about BC, perceived individual risk and comparative risk. A structural equation modelling (S.E.M.) was built to investigate the relationship between illness perceptions and perceived individual risk.

**Results:**

Participants reported negative illness perceptions about BC The individual risk perception and the compared risk perception for BC were low. Participants with a family history of BC reported more negative illness and higher risk perceptions. The relationship between illness perceptions and perceived individual risk was significant and mediated by compared risk. The S.E.M. explained 55.9% of the variance in predicting perceived individual risk for BC.

**Conclusion:**

Women's views of BC are important factors in risk perception and may provide culturally sensitive clues to promote early screening for BC in Arab countries. This may be important for policymakers to design intervention strategies to lower health risks, considering the different ways in which women perceive their risks for BC.

## Introduction

Breast cancer (BC) is among the most common life-threatening public health problems of global concern. According to the World Health Organization [1], BC is the most frequent cancer among women, impacting 2.1 million women each year, causing the highest number of cancer-related deaths. According to the same source, it is estimated that in 2018 BC accounted for approximately 15% of all cancer deaths among women.

In the last 20 years, several published epidemiological reports in different parts of the world show a significant increase in the BC mortality rate [2]. In the Middle East region, the incidence of BC has substantially increased in the last years. It is the most common cancer among Arab women in Eastern Mediterranean Region (E.M.R.) [3], especially those younger than 50, and the incidence is expected to double by 2030 [4]. According to the International Agency for Research on Cancer, from the Global Cancer Observatory from the World Health Organization, the number of new cases of BC in the U.A.E. in 2018 was 1054 (39.9%), the incidence was 22.4%, and the mortality 12.4% [5], being the most frequent type of cancer in women. Previous studies referred that women are diagnosed at later stages in the Middle East, and a significant proportion of the diagnosis are women in their thirties and forties. The average age of BC presentation is one decade earlier than in western countries [6, 7]. Considering the higher relevance of BC in the Middle East, it seems important to consider how healthy women perceive BC and what factors may contribute to their risk perception.

It has been an increased focus on how healthy people think about health and illness and how their individual beliefs relate to health-related behaviors and risk perception [8]. There is extensive literature on illness perceptions and how these views impact coping and illness outcomes. The most widely studied theoretical model of illness perceptions has been the Self-Regulation Model (SRM), also called Common-sense Model (CSM), developed by Leventhal et al. (1980). Illness perceptions guide the response to an illness threat and activate coping procedures to deal with the threat [9–12].

How women manage an illness threat may well depend on their perceived susceptibility to that specific threat. Perceived susceptibility (or perceived vulnerability, or perceived risk) is also central to Leventhal's parallel process model (1980). It posits that perceived susceptibility to a disease may trigger either danger control, related to health behaviors to mitigate the threat, or fear control, concerning actions to reduce negative emotions associated with perceptions of vulnerability [13]. A recent systematic review has supported the validity of the illness representation construct in the CSM. by summarizing the associations between illness perceptions, coping behaviors, and illness outcomes in people with cancer [14]. This review found, for illness perceptions, small to moderate relationships with coping behaviors and moderate to large relationships with illness outcomes. A recent study about women's illness perceptions of BC in Turkey concluded that the meaning of illness from patients with BC should be addressed and treatment planned accordingly [15]. This is consistent with a previous systematic literature review on illness perceptions about BC, supporting the need for healthcare providers to consider these perceptions in their usual care [16]. These findings suggest that cognitive representations are critical in understanding individual responses to cancer in patients and non-clinical samples.

More recently, it has been argued that risk perception could be understood as part of a self-regulatory process [12] in which illness perception dimensions can influence risk perceptions and their relationship with behavior. Although earlier research has found that illness perceptions can influence risk perception [17], the relationship between illness perceptions and perceived risk has been less explored in BC. Most studies have examined illness-specific beliefs and behaviors within specific groups of patients or groups at risk from a particular illness [18]. Less attention has been given to healthy groups of the population, considering how individual beliefs about health and illness may influence risk perception (perceived susceptibility to disease). Research suggests that psychological factors also play a role in people's judgments of vulnerability [19, 20]. Psychological factors influencing BC's risk perception include the perceived nature and the heuristic processing of BC as a health threat [21]. According to these authors, processing a threat involves two cognitive functions: availability heuristic and representativeness heuristic. The former is the influence of the ease with which examples of an event come to mind in judging the probability of an event [19]. This easiness increases the availability of the threat and, as a result, its perceived likelihood [22]. The latter involves judging the probability of an event by its similarity to events with comparable features (e.g., the belief that one is similar to the type of person who develops a specific disease) [23]. Specifically, the perceived similarity to the person who develops BC is the strongest correlate of perceived BC risk, followed by the perceived prevalence of BC and the proximity of a relative/friend with BC [13]. As such, two commonly used measures of perceived susceptibility have been comparative risk and absolute risk (perceived individual risk) [21]. To the best of our knowledge these measures have not been explored in Arab women concerning the risk of BC. Risk research about BC has considered variables such as family history and age, but less attention has been given to other possible factors such as illness perceptions in healthy women. Perceptions about BC may also cause worry in healthy women considering their risk of developing the disease [24]. Their findings indicated that risk perceptions partially mediated the relationship between illness perceptions and worry in healthy women. Consequently, understanding possible psychological factors and mediators of BC's perceived risk is an essential focus of interest.

Although there is a considerable body of research about BC in Arab countries, research has been mainly on patients. Other studies have focused on knowledge and screening beliefs and preventive practices for BC [7, 25–27]. However, the focus on illness perceptions about BC in this region has been less explored. A recent review described the experiences of women with BC in Arab countries, showing that these experiences are influenced by the women's and society's views of cancer, the women's role in society and family, religious faith and the healthcare context, and access to treatment choices and information [28].

Another important aspect of Arab society is the incursion of individualist healthcare norms into a collectivist society where the family and their needs and goals take over personal needs [29]. Decisions regarding individual health are a matter for the extended family, often directed by the males (husband, father, and brother) [30]. It is essential to understand these traditional beliefs to grasp the cultural aspects of early cancer detection to reduce morbidity and mortality, resulting in more effective treatment regimens and better survival rates [31]. Also, the way that the cultural aspects of early cancer detection can influence risk may promote optimistic biases by comparison to their close relatives or friends, influencing individual risk estimation and self-efficacy beliefs. Findings on risk perception of Turkish women in primary care showed a high optimism and a low-risk perception [15]. Previous evidence showed that many women overestimate their BC risk, showing an inappropriate risk perception preceded by inappropriate health behavior [21]. This is key to understanding the underlying mechanisms to plan interventions to promote preventive practices. A more accurate public awareness about risk perception for BC may increase the uptake of early detection screening. Raising awareness and improving knowledge about the disease will reduce the number of cases at later stages of the disease [32]. Moreover, a better understanding of possible determinants of risk perception for BC in young women can be a component of informed decision-making to expand the scope of preventive actions. Given the scarcity of studies about BC in healthy women in the U.A.E., this study aims to further understand which psychological factors may contribute to the perception of risk for BC.

In the present study, our first goal was firstly to assess illness perceptions of BC, and risk perceptions (comparative risk and perceived individual risk), in a sample of young, healthy women in the U.A.E. Our second goal was to explore whether illness perceptions and compared risk predict individual risk perception for BC in healthy young women in the U.A.E..We hypothesized that illness perceptions about BC are significantly associated with risk perception, and that relationship can be mediated through comparative risk. Identifying which illness-related beliefs are associated with perceived risk highlights key variables in forming perceived individual risk that may inform preventive measures and interventions for health-protective behavior.

## Methods

### Procedure

A cross-sectional online survey was conducted from February to May 2020 at Zayed University in the U.A.E. Participation was voluntary and anonymous. Informed consent was obtained from all the participants. The study received ethical clearance from the research ethics committees of Zayed University (ZU19_107_F). All methods were performed following the relevant guidelines and regulations.

### Participants

Given the rationale for the present study in the Arab region (average age of BC presentation is one decade earlier than in western countries), we used a convenience sample of young adult women studying at the university. Two hundred and ninety-eight female students from different courses completed an online survey sent through a Campus announcement. The mean age was 20.1 years old (sd = 1.7), ranging from 18-to 25 years old. Concerning marital status, 91.3% of the students were single, and 8,5% were married. Twenty-one percent of the sample reported a family history of BC.

### Measures

The measures included socio-demographic data (age, marital status), illness perceptions, and risk perceptions (comparative risk and perceived individual risk).

#### Socio-demographic information

The information gathered included age, marital status, and family history of BC For the comparative analysis regarding socio-demographic information, two age groups [18–20] and [21–25] were created using the median as a cutoff for younger and older participants. Family history was measured as yes/no.

#### Brief-illness perception questionnaire (Brief-IPQ)

The illness perceptions were measured using an adapted experimental English version of the brief-IPQ [33] for a non-patient sample. The B-IPQ has been widely used across several illnesses and ethnic groups, being translated into 26 languages. Furthermore, it showed good psychometric properties across many studies [34]. An English version was used since the University courses are taught in English. This brief scale has been used in several studies to assess illness perceptions in patients[35, 36]. The Brief-IPQ uses a single-item scale approach to assess perceptions on a continuous linear scale. It consists of eight items rated on a scale from 0 (minimum) to 10 (maximum). The items assess cognitive perceptions such as effect on life or consequences (item 1); duration of illness (item 2); control over illness (item 3); beliefs about the effectiveness of treatment (item 4); and experience of symptoms (item 5). Items 6 and 8 assess emotional aspects, including concern about illness and emotional reaction to the illness. Item 7 assesses the degree of understanding of the illness (Coherence). The causal attributions are assessed with an open question. The participants answered what they believe are the main causes of BC.

#### Risk perceptions

These measures were adapted from previous studies on risk perception in non-clinical samples (Figueiras et al., 2017). Individual risk perception was measured, asking participants to rate their perceived risk of developing BC in the next 10 years. Comparative risk perception was measured, asking participants to rate their perceived chance of developing BC compared to a woman of the same age from 0 (not at all at risk) to 10 (very high risk)*.* The scale was inspired by previous literature on verbal probability expressions used to communicate risk [37]. Both types of measures were used to research the correlates of perceived susceptibility [13]. Previous evidence showed that using a single item approach is not empirically disadvantageous compared with multiple items self-report measures [38].

### Data analysis

**Fig. 1:**
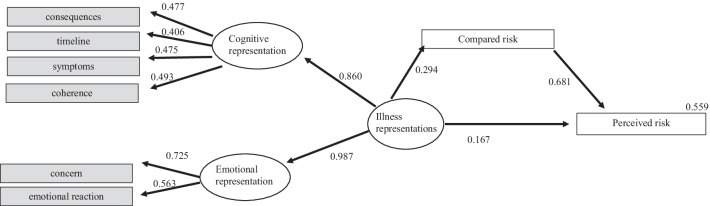
Illness perceptions and compared risk as predictors of individual risk perception for BC

The data analysis was performed in two steps. First, we used a descriptive approach to assess illness perceptions, comparative risk and perceived individual risk. The five causes most mentioned were described in descending order. Differences related to age groups and family history were studied using t-tests for large samples under the assumption of the normal distribution of means as per the Central Limit Theorem. Using Pearson correlations, we also explored the correlations between the dimensions of illness perceptions and those with the risk variables. Despite the non-normality of data, parametric analyses have been known to produce reliable results even when data are moderately non-normally distributed [39]. These statistical analyses were performed with IBM SPSS version 26 [40]. Power calculations were performed (post-hoc) using Gpower3.1 [41]. To compare the two independent groups of age and family history, post hoc power was calculated for a medium effect size to reduce type I error probability. Due to the low percentage of married participants, marital status was not considered for analysis. The hypothesized structural model of BC perceived risk (see Fig. 1) was fitted with the lavaan package (v. 0.6; [42]) for the R statistical system. Estimation was carried out using robust maximum likelihood estimation since several observed variables deviated from the normal distribution (skewness ranged from − 9.745 to 4.319; kurtosis ranged from − 0.937 to 0.646). Goodness of fit for the model was assessed with the robust versions of the Chi-square statistics, Goodness of Fit Index (G.F.I.), Tucker-Lewis Index (TLI), Root Mean Square Error of Approximation (RMSEA), and Standardized Root Mean Squared Residual (SRMR). Fit was judged adequate for CFI and TLI above 0.9 and RMSEA and SRMR below 0.08 [43, 44].

## Results

### Descriptive analysis

The first goal of the present study was to assess illness perceptions of BC and risk perceptions, including differences according to the family history of BC and age groups. The descriptive analysis of the study variables is depicted in Table 1. The participants reported strong negative beliefs about BC's consequences, a moderate negative belief in a chronic timeline (duration), the experience of symptoms, and personal control over BC. There is a positive belief about the effectiveness of the treatment to control BC. There is a moderate negative belief concerning emotional representation and concern, indicating negative emotions associated with BC. There is an average level of comprehension of BC regarding causal attributions. The five reasons most often mentioned were genetics (52.7%), lifestyle (30.2%), diet (18.8%), family history (18.5%) and radiation (13.4%). Concerning risk perception, the participants reported low-risk perception and low compared risk.

**Table 1:** Comparison between groups by age group and family history of BC—t-test for independent groups (N = 298)

Variables	Total	Differences by age group			Differences by family history		
		Younger (n = 186)	Older (n = 112)		Yes (n = 64)	No (n = 234)	
*Illness percept. (0–10)*	M (SD)	M (SD)	M (SD)	Signif	M (SD)	M (SD)	Signif
Consequences	8.1 (2.21)	8.0 (2.24)	8.3 (2.14)	ns	8.7 (1.65)	8.0 (2.31)	0.008
Timeline	6.0 (1.80)	5.7 (1.61)	6.5 (1.98)	0.001	6.7 (1.79)	5.8 (1.75)	< 0.001
Identity (symptoms)	6.1 (2.56)	6.0 (2.55)	6.3 (2.58)	ns	6.0 (2.73)	6.2 (2.52)	ns
Treatment control	3.2 (2.30)	3.1 (2.23)	3.2 (2.42)	ns	3.3 (2.46)	3.1 (2.26)	ns
Personal control	5.6 (2.37)	5.8 (2.34)	5.1 (2.35)	0.018	5.6 (2.37)	5.6 (2.37)	ns
Coherence	5.0 (2.54)	5.2 (2.42)	4.6 (2.68)	ns	4.4 (2.49)	5.1 (2.54)	ns
Concern	6.5 (2.70)	6.4 (2.70)	6.8 (2.68)	ns	7.3 (2.56)	6.3 (2.70)	0.009
Emotional reaction	6.4 (2.93)	6.1 (2.84)	6.8 (3.04)	0.044	6.9 (2.84)	6.2 (2.95)	ns
*Risk Perception (0–10)*							
Perceived individual risk	3.4 (2.48)	3.3 (2.43)	3.7 (2.55)	ns	4.7 (2.84)	3.1 (2.26)	< 0.001
Compared risk	3.3 (2.51)	3.2 (2.48)	3.5 (2.55)	ns	4.2 (2.84)	3.0 (2.35)	0.003

*Note.* ns = not significant

#### Group differences for risk, and illness perceptions

According to age group, there were significant differences in some dimensions of illness perceptions about BC. Younger women hold beliefs concerning a shorter duration, less negative emotional representation, and stronger beliefs about personal control over BC (Table 1). According to the family history of BC, there were significant differences in illness perceptions, namely, consequences, timeline, and concern. There were also significant differences in individual perceived risk and compared risk. Participants with a family history of BC reported more negative beliefs about BC's consequences, a more chronic timeline, greater concern, and a higher individual, and compared risk perception compared with the group with no family history of BC. For the comparisons between age groups and family history, 99,4% and 97% post hoc power were achieved for a medium effect size, increasing the confidence that true effects were detected.

#### Pattern of correlations between illness perceptions dimensions and risk perceptions

The pattern of correlations between illness perception dimensions and risk perceptions is depicted on Table 2. There were significant correlations between several illness perceptions dimensions. Identity (patient experience of symptoms) was significantly associated with a chronic timeline, lower personal and treatment control, more severe consequences, a stronger negative emotional reaction, higher scores of concern and a lower understanding of BC. The emotional dimensions (concern and emotional reaction) were significantly associated with the perception of the experience of symptoms, more serious consequences, a chronic timeline, a higher concern and lower comprehension of BC. The correlations of illness perceptions dimensions and risk perceptions (comparative and perceived risk) indicated that higher compared risk was negatively associated with the level of understanding about BC and positively associated with the emotional dimensions. Higher comparative and perceived individual risk perceptions were associated with more negative emotional representations about BC.

**Table 2:** Pattern of significant correlations between illness perceptions dimensions and risk perception (n = 298)

*Illness perceptions*	1	2	3	4	5	6	7	8	9	10
1. Identity										
2.Consequences	0.22***									
3. Timeline	0.20***	0.27***								
4. Personal control	− 0.14**	0.016	− 0.069							
5. Treatment control	− 0.23***	− 0.077	0.12*	0.062						
6. Coherence	− 0.30***	− 0.13*	− 0.18**	0.16**	0.18**					
7. Emotional reaction	0.23***	0.29***	0.17**	0.042	− 0.12*	− 0.26***				
8. Concern	0.27***	0.33***	0.24***	0.087	− 0.037	− 0.34***	0.37***			
*Risk perceptions*										
9. Compared risk	0.031	0.041	0.075	− 0.063	− 0.009	− 0.14*	0.23***	0.22***		
10. individual perceived risk	0.054	0.054	0.061	0.043	0.022	− 0.11	0.29***	0.30***		

^*^p < 0.05; **p < 0.01; *** *p* < 0.001

### Psychological factors associated with perceived risk of breast cancer

The second goal of the present study was to explore whether illness perceptions dimensions and compared risk predict individual BC risk perception in healthy young women in the U.A.E.. A SEM was built to investigate the hypothetical relationship between illness perceptions and compared risk as factors associated with individual risk perception for BC. The model tested the influence of illness perceptions and comparative risk on perceived individual risk perception. More specifically, we sought to test whether comparative risk could mediate the relationship between illness perception dimensions and perceived individual risk.

The proposed BC risk model showed a good fit to the data (χ^2^[18] = 34.131, *p* = 0.012, CFI = 0.965, TLI = 0.945, RMSEA = 0.055, *p* = 0.355, SRMR = 0.049). No modification indices were used to improve fit, but the variance of emotional representations was constrained to 0.1. Overall, the model explained 55.9% of the perceived risk (r2 = 0.559, *p* < 0.001). Illness perceptions had a standardized direct effect of 0.167 (*p* = 0.024) and a standardized indirect effect mediated by the compared risk of 0.200 (*p* = 0.004). The total standardized effect of illness perceptions on the perceived risk was 0 0.367 (*p* = 0.003). Figure 1 displays the standardized estimates for the cancer risk model.

## Discussion

The present study investigated illness perceptions about BC and the perceptions of comparative risk and perceived individual risk in a sample of young Emirati women. The participants hold a common-sense model of BC composed of beliefs about the patient's experience of symptoms, serious consequences, high concern, chronic timeline, and a negative emotional representation, although showing a strong belief in the treatment's efficacy, a moderate belief in personal control, and a moderate level of understanding. Moreover, the pattern of the intercorrelations showed between the illness perceptions dimensions was consistent with previous studies [45, 46], showing a "coherent" pattern of association between the dimensions. The descriptive analysis regarding causal attributions showed that the top causes were genetics, followed by lifestyle, diet, family history and radiation. This analysis is speculative since we have no previous comparative evidence from the U.A.E.. However, these causal attributions raise some questions regarding how genetics can be understood as family history and lifestyle can be considered amenable to change. Further research is needed to clarify how perceptions of causal attributions can influence preventive behavior. This may be associated with the availability of information about BC shared in the community and the level of education of the participants. The significant association between the level of concern and the emotional dimensions of the illness representation with risk perception suggests that lay beliefs about BC may be interconnected with perceptions of vulnerability, mainly focused on emotional representations of BC. Also, it may suggest that healthy groups of the population hold common-sense models of BC. Specific beliefs associated, independent of the direct experience with the illness, may influence the adoption of preventive behaviors for BC. The compared and perceived individual risk were perceived as low. There is evidence that women's risk for developing BC increases with age [21]. Being a non-clinical sample, and given the age range, one might expect that perceived vulnerability for BC is considered low. Furthermore, the CSM advocates that these perceptions are dynamic and may integrate new information from the socio-cultural context [47]. Illness perceptions about BC may change, influencing the perceived individual risk through the life span. Longitudinal studies analyzing changes in these perceptions may inform future interventions to promote BC's preventive behaviors.

There were some significant differences by age group, indicating that younger women hold more positive beliefs regarding timeline, personal control, and emotional reaction. This highlights the need to consider socio-demographic variables regarding illness perceptions and their possible influence on risk assessment. Recent research concerning breast and cervical cancer screening in four Gulf Cooperation Council countries has shown that socio-demographic characteristics were associated with levels of screening [48]. A better understanding of possible determinants of risk perception for BC in young women can be a component of informed decision-making to expand the scope of preventive actions. Participants with a family history of BC hold more negative beliefs about the disease and reported a higher risk perception. These findings were similar to other studies [13, 49], in which family history influenced women's risk perception. Having a family history of BC may raise awareness and improve knowledge about the disease to reduce the number of cases diagnosed at later stages of the disease [32].

The illness schema about BC show similarities with a previous study with healthy women [24] and the pattern of association between illness perceptions of BC and risk perception. To the best of our knowledge, no previous studies looked at illness perceptions of BC and risk perception in healthy women in the U.A.E.. We sought to explore a model by identifying variables that potentially may contribute to the formation of perceived risk for BC in a sample of Emirati healthy women. We consider that the present findings raise some essential questions concerning how individual illness perceptions may be shared in the community and how they may contribute to risk awareness and preventive behaviors, such as early screening. Moreover, given the characteristics of the culture, their illness perceptions can be influenced by spiritual or religious beliefs, as found in a study about the perception of health states [50]. The extent to which religious beliefs may influence illness perceptions is still unexplored. It may be important to further understand how religious beliefs can influence beliefs about consequences, personal control, or treatment of BC in healthy women to address prevention and early screening practices. Since perceptions of control were unrelated to risk perception, perhaps other sets of beliefs are likely to contribute to the perceived vulnerability to BC. This deserves further investigation in the context of Arab women. Another issue relates to the illness perceptions of significant others (family members) and how they can influence screening and early detection. Previous studies in the region highlighted the role of culture as a barrier for early detection, considering attitudes towards gender roles and expectations, conservative contexts, or lack of support from male relatives [26].

The present findings suggest that BC's beliefs might have been influenced by social and cultural factors and how women deal with some health issues. Also, social messages might have specific circuits within family members and influence perceptions about an illness and risk perception. Women may create their illness perceptions and risk perception by integrating information shared in the community and between close family members with information derived from personal experiences. This, in turn, influences how they make sense of it and make decisions to seek help when needed.

A large body of research shows that risk perception is a subjective psychological construct influenced by cognitive, emotional, social, cultural, and individual variation between individuals and between different countries [51]. Although risk perception is commonly defined as absolute personal risk, it is inherently comparative because people need to have some parameters to assess their own risk [52]. The role of comparative risk as a mediator between illness perceptions and perceived individual risk suggests that people use comparative risk to understand and judge their safety. Individuals are sensitive to any form of comparison between their own risk and their peers' average risk. The available information about the illness, individual level of understanding, and psychological variables influence comparative risk (e.g., social comparison) [53]. Given the characteristics of the close interactions between women in Arab countries, it is likely that comparative risk may influence their risk perception, which may have implications for BC screening. This deserves further investigation, given the possibility of a risk bias that may influence BC's risk perception and subsequent preventive behavior.

The present study has some limitations. Due to convenience sampling, the generalizability of the findings is limited. The participants were university students, probably with higher knowledge and understanding of health issues. However, we consider that this population can be particularly important because they can be potential sources of information for their families and the general population. The measures used were in English, although the participants seem to be familiar with this language. Since the focus of the study was on young ages, the risk perception measure was focused on a closer time frame (next 10 years) and did not explore lifetime risk perception. Middle East women are diagnosed on average 10 years earlier than in western countries. Moreover, socio-demographic and religious beliefs should be considered in future research to further explore their influence on perceived vulnerability to cancer. We did not inquire about screening or self-breast examination practices due to the possibility of social desirability responses and avoiding questions that can be considered intrusive. Characteristics of social and economic status and religion were not included because they were considered too personal and sensitive for this population.

Despite these limitations, the benefits of this study's findings are potentially far-reaching and contribute to a new approach to beliefs about BC and its implications for risk perception in Arab women. The previous literature has focused on beliefs and behavior. The present study focused on the strength of the illness-related beliefs as a predictor of perceived individual risk and the mediating role of comparative risk.

## Conclusions

The findings suggest that illness perceptions about BC are associated with perceived risk for BC in healthy women. Also, they highlight the need to consider the mediating role of comparative risk as a potential source of risk bias. This is essential for the cultural understanding of these processes, given the differential role of social comparison in different contexts. This may have important implications for decision-making about screening behaviors for BC, considering the beliefs about what others do or the extent they share the same characteristics. Strategies such as using social comparison nudges [54] (SCNs) can be useful by providing these women with information about the behavior of relevant peers since this information can influence their behavior.

The present findings contribute to raising awareness to deliver campaigns aimed at screening participation for BC. These should also consider illness and risk perceptions of significant relatives as important influences in seeking health care. This may be important for policymakers to design intervention strategies to lower health risks, considering the different ways in which women perceive their risks for BC. Addressing how women's views about BC may influence their concerns and risk perception may impact guidelines for risk communication. They could consider what can be culturally appropriate to meet the health care needs of women for early screening and preventive activities for BC in the Middle East.

## Data Availability

The dataset generated during and analyzed during the current study is not publicly available due to the sensitive nature of the topic in this cultural context, but is available from the corresponding author on reasonable request (maria.santos@zu.ac.ae).
